# The Nation's Awakening: An Opportunity for the Churches

**Published:** 1916-01-01

**Authors:** 


### The Hospital

The Workers' Newspaper of Administrative Medicine and Institutional Life, Administration, National Insurance and Health.

No. 1542, Vol. LIX. SATURDAY, JANUARY 1, 1916. PRICE ONE PENNY.

### THE NATION'S AWAKENING: AN OPPORTUNITY FOR THE CHURCHES.

At the church we attended on Christmas Day the preacher took for his subject "The Irony of Christmas, 1915." The point he laboured to make was that the majority of people regarded Christmas Day, and everything connected with it, having in view the great war and all that has happened since that war began, as a mockery, for all these things were utterly opposed to Christmas, to the birth at Bethlehem, and to the teaching and life example of Christ Himself. It is difficult enough for the cleverest and most capable mind to realise present problems, and it is certainly above the capacity and grasp of ordinary people to apprehend completely the vast questions thus raised, and their real bearing upon the actual conditions of to-day. There is for the superficial observer no peace, nor the appearance of peace on earth, owing to the waging of some nine wars in so many portions of the civilised world. This, however, is a casual view, which ignores altogether the teachings of life, the progress of the nations, the raising of the moral standard of the world, and the effects of Christ's teaching and example with the results which have flowed from them.

If we take the position of our own country to-day, realise what was the actual condition of the people in these islands, and their attitude and habits in regard to Christmas in 1913, and compare them with what they were on December 25, 1915, the whole fabric on which the charge of irony rests is blown to pieces. For the first time after upwards of forty years' residence in London, a tour of many portions of the metropolis, and a study of the habits and proceedings of the people on the 25th December last, afforded evidence everywhere of the great fact, that, this day was universally regarded and held as a family festival, a home day. All who were present were in the closest touch with those who were absent. The occasion was one when every man and woman displayed a sense of the knitting together of the whole nation and Empire by an united resolve to fight to the death to uphold Christ's teaching and principles against a pagan, a brutal, and a diabolical tyranny. At the beginning of the war some representative women who had given of their best, in the shape of husbands, or sons, or both, to this cause, declared, that should disaster befall their dear ones, they would decline to wear mourning, and that if they had to wear anything distinctive it must be white, in testimony of the glorious fact, as the Bishop of London put it, that death on the battlefield, in defence of the principles for which the Allies are fighting, was the best preparation a man could wish to have for his entrance into the Great Beyond.

In fact, so far from it being accurate to describe the actual position of affairs, and its effects upon our population and people, as the irony of Christmas, it should be the preacher's privilege with absolute conviction to point out, that nothing that has happened to this country in the lifetime of any living person has done so much as has this war to make Christmas, 1915, mean life, and hope, and renewed strength, the uplifting of the character and the gathering together of families and communities throughout these islands and throughout the Empire. Turn where you will, analyse and study the actual effects upon the people of Christ's teaching in the light of the lessons taught and the conviction excited amongst them by this great war, and every man and woman amongst us has the most convincing cause to thank God and take courage.

Christmas Day, 1915, was, in a sense probably never before equalled in the history of our nation, the day of each family—the home day—when everyone felt they could best do their part in these anxious times by gathering together, standing unitedly with the conviction that the way to find strength and to prepare for the future was for each one to do his best to cheer the other and to spend this day together in rational enjoyment. So Londoners spent it under influences of the highest standard of individual conduct and effort of which they were capable. All thought of self was absent, owing to a common thirst for the humanising influences which flow to those, who devote a measure of their time to cheer and brighten the lives of their neighbours. This was the more remarkable because the very vastness of London tends to destroy neighbourliness, and its ordinary working-day pursuits to force inwards and oppress individual aspiration. The war seemed to alter all this on Christmas Day, the natural man and woman gave free vent to strong, pure, healthy, neighbourly feelings, with results, which in God's good providence, may prove fruitful as an example to the world.

